# Unique Strain of Crimean–Congo Hemorrhagic Fever Virus, Mali

**DOI:** 10.3201/eid2005.131641

**Published:** 2014-05

**Authors:** Marko Zivcec, Ousmane Maïga, Ashley Kelly, Friederike Feldmann, Nafomon Sogoba, Tom G. Schwan, Heinz Feldmann, David Safronetz

**Affiliations:** National Institutes of Health, Hamilton, Montana, USA (M. Zivcec, A. Kelly, F. Feldmann, T.G. Schwan, H. Feldmann, D. Safronetz);; University of Manitoba, Winnipeg, Manitoba, Canada (M. Zivcec, H. Feldmann);; University of Sciences, Techniques and Technologies of Bamako, Bamako, Mali (O. Maïga, N. Sogoba)

**Keywords:** Nairovirus, Bunyavirus, tick-borne, West Africa, viral hemorrhagic fever, field studies, CCHF, Crimean–Congo hemorrhagic fever virus, CCHFV, viruses, humans, ticks Hyalomma ticks, Mali

**To the Editor:** Crimean-Congo hemorrhagic fever (CCHF) is an acute viral infection that causes mild to severe hemorrhagic fever characterized by petechiae, ecchymosis, disseminated intravascular coagulation, and multi-organ failure (*1*). The etiologic agent, CCHF virus (CCHFV; family *Bunyaviridae,* genus *Nairovirus*), is maintained in enzootic cycles involving agricultural and wild animals and the vector, *Hyalomma* ticks. (*2*). CCHF predominantly affects persons who have 1) substantial contact with ticks and/or agricultural animals in areas where CCHF is endemic or 2) close contact with infected persons, predominantly close relatives and health care workers. The case-fatality rate for CCHF is generally accepted as 30% (*1*).

CCHF has a wide geographic distribution; cases have been reported in >30 countries across Africa, southeastern Europe, the Middle East, and western Asia. In the western African countries of Nigeria, Mauritania, and Senegal, serologic evidence of CCHFV infections in humans and agricultural animals has been documented frequently (*3*–*5*); however, reports of the disease in humans have been limited to Senegal and Mauritania (*6*,*7*). In neighboring Mali, where the tick vector is known to be present, little information exists regarding the presence of CCHFV. Thus, to determine if the virus is circulating undetected in Mali, we conducted a study to determine if CCHFV is present in *Hyalomma* ticks in the country.

In November 2011 and March 2012, unfed *Hyalomma* ticks (adults and nymphs) were collected from 20 cattle at the Daral livestock market (12° 49.855′ N, 08° 05.651′ W) near the town of Kati, Mali, ≈25 km from the capital, Bamako. In the field, ticks were visually identified to genus and pooled accordingly (3–4 ticks per pool, all collected from the same animal). A total of 23 tick pools, representing 80 ticks, were manually homogenized, and RNA was extracted and tested for the presence of CCHFV RNA by using in-house assays that selected for 3 virus genes. Of the 23 tick pools tested, 1 was positive for CCHFV by all 3 assays. Phylogenetic analysis of the complete nucleocapsid protein gene (KF793333) showed that the CCHFV strain from Mali most closely resembled a strain from Mauritania (GenBank accession no. ArD39554), sharing 98% sequence identity (Figure, panel A). 

**Figure:**
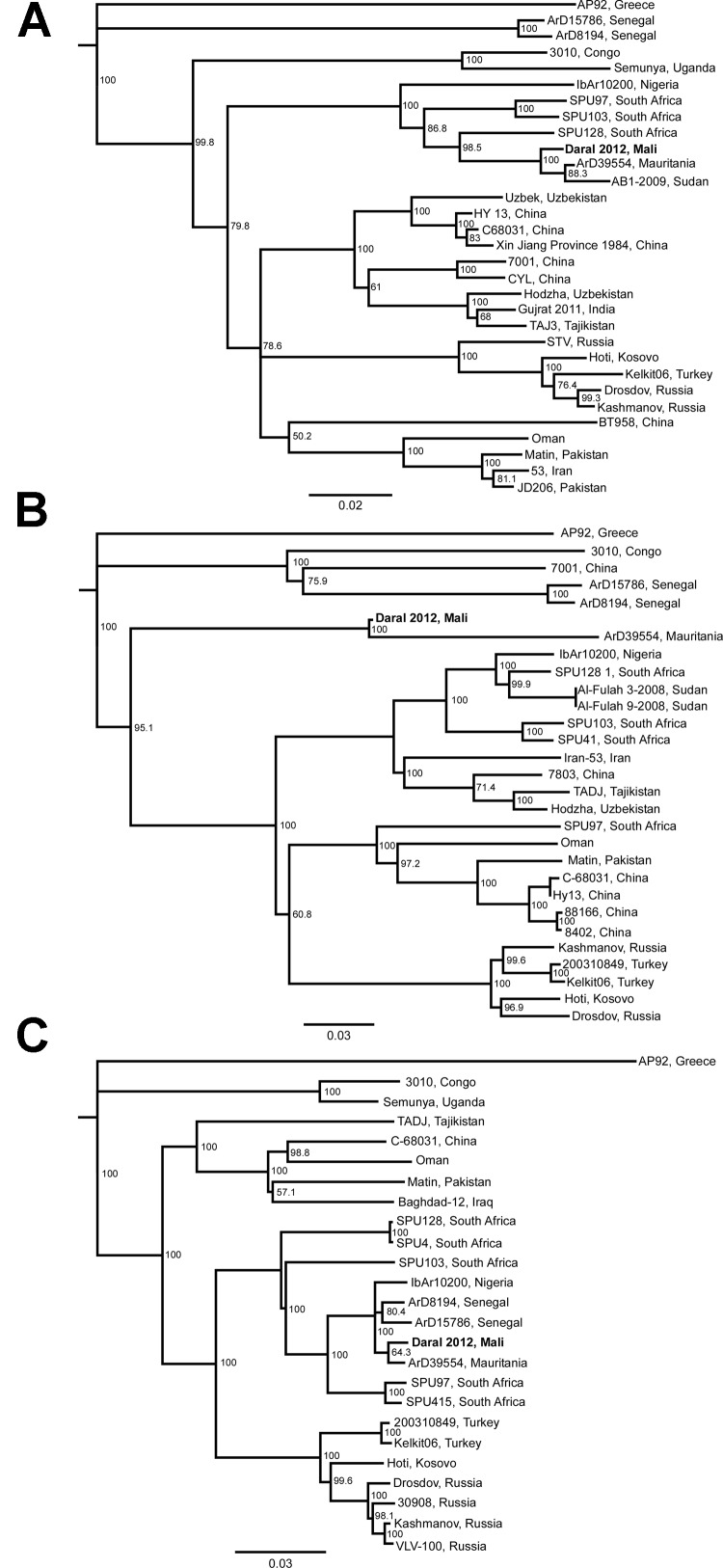
Phylogenetic analysis of Crimean–Congo hemorrhagic fever virus (CCHFV) was conducted on the complete nucleoprotein (small genomic segment, nt ≈50–1,500) (A), a 900-bp fragment of the glycoprotein precursor (medium genomic segment, nt ≈4190–5060) (B), and a 1,200-bp fragment of the viral polymerase (large genomic segment, nt ≈590–1760) (C). The fragments were amplified from pooled ticks, and sequence analysis was conducted by using ClustalW (www.ebi.ac.uk/Tools/msa/clustalw2/). Trees were constructed by using the Jukes–Cantor neighbor-joining method with bootstrapping to 10,000 iterations and compared with published sequences of full-length small, medium, and large segments. Bold indicates CCHFV strain from *Hyalomma* ticks that were collected from cattle at the Daral livestock market near the town of Kati, Mali. Scale bars indicate substitutions per site.

Further analysis of fragments of the medium segment (pre-Gn coding region, KF793334) and large segment (polymerase coding region, KF793335) confirmed these findings, showing sequence identities of 91% and 98%, respectively, with ArD39554 (Figure, panels B, C). In a Biosafety Level 4 facility at Rocky Mountain Laboratories, Hamilton, Montana, USA, the original homogenates from the positive pool were passaged in multiple cell lines. After 3 passages, no discernible cytopathic effect was observed and, aside from the initial passage, CCHFV RNA was not detected.

Genetic identification of ticks in the CCHFV RNA–positive pool was conducted as described (*8*,*9*). Amplified sequences most closely resembled those of *H. dromedarii*, (97.2%–100% sequence identity), although genetically, we cannot exclude the possibility that *H. truncatum* and *H. rufipes* were present with individual sequence identities of >97% to published sequences.

The Daral cattle market in Kati is the largest of its kind in Mali, and animals from across the country come into the market every week. Although the market provided a convenient opportunity for collecting ticks, we cannot determine where the infected ticks, and possibly cattle, contracted CCHFV because the animals traversed great distances on foot before arriving at the market. Nevertheless, this study demonstrates the presence of a distinct strain of CCHFV in *Hyalomma* ticks in Mali, thereby expanding the geographic distribution of this virus in western Africa. Not surprisingly, the highest sequence identity for the CCHFV strain from Mali is to strains known to circulate in neighboring countries (*10*). We propose Daral 2012 Mali as the temporary designation for this sequence. Unfortunately, our attempts to isolate the virus were unsuccessful, most likely because of processing and storage conditions for homogenates used in these studies.

Species of *Hyalomma* ticks are widely distributed across western Africa, and although reports of CCHF are limited to a few countries, CCHFV is most likely circulating undetected in vast areas of this region. No cases of CCHF have been reported in Mali; however, on the basis of our findings, the potential for human infections exists. Thus, CCHF should be considered in the differential diagnosis of febrile illnesses, with or without hemorrhagic symptoms, in residents of Mali and for persons with a recent history of travel to this country.

The ease of CCHFV transmission and the high case-fatality rate associated with infection could have a potentially substantial effect on public health. Future studies in Mali are required to define the geographic distribution of infected ticks and animals and to isolate CCHFV to help focus public health preparedness and countermeasures. In addition, across Mali, operational protocols should be reviewed for persons working at jobs in which the risk for CCHFV transmission is high (e.g., occupations with direct contact with agricultural animals and/or animal blood products), and appropriate countermeasures should be put in place to prevent transmission among such persons.
